# The Correlation of Computed Tomography (CT)-Based Body Composition and Survival in Pancreatic Cancer Patients: A Systematic Review

**DOI:** 10.3390/tomography12010008

**Published:** 2026-01-08

**Authors:** Lena Supe, Stefania Rizzo

**Affiliations:** 1 Faculty of Biomedical Sciences, Università della Svizzera italiana, 6962 Lugano, Switzerland; 2 Clinic of Radiology, Institute of Integrated Diagnostics of Southern Switzerland (IDISI), Ente Ospedaliero Cantonale (EOC), 6900 Lugano, Switzerland

**Keywords:** body composition, sarcopenia, adiposity, pancreatic cancer, survival

## Abstract

**Background/Objectives**: Pancreatic cancer is among the most aggressive malignancies, with poor survival rates. Emerging evidence suggests that body composition, including skeletal muscle mass and adiposity distribution, plays a crucial role in predicting patient outcomes. However, its impact on survival in pancreatic cancer remains incompletely understood. The aim of this systematic review was to assess the correlation between body composition parameters and survival outcomes in pancreatic cancer patients, focusing on overall survival. **Methods**: A comprehensive literature search was conducted, including three main components: pancreatic cancer, body composition, and survival outcomes. **Results**: 23 studies were included in this review. The findings indicate that body composition can serve as a predictor of survival in pancreatic cancer patients, with 21 studies reporting a significant correlation. The most frequently observed predictor, with 11 studies reporting, was not a baseline parameter but rather changes in parameters over time during treatment. However, discrepancies remain regarding the extent of predictive power and the relative importance of individual components. **Conclusions**: Specific body composition parameters hold potential as prognostic indicators of survival in pancreatic cancer patients. However, further research is necessary to establish consistent patterns and to clarify which parameters are most predictive and under what conditions.

## Simple Summary

In pancreatic cancer, the amount and distribution of muscle and fat can influence how well the patient does after surgery and during treatment. Standard measures like body mass index do not reliably predict survival, whereas detailed assessments of body composition using CT scans may provide more accurate prognostic information. Patients with higher levels of visceral fat (fat around abdominal organs) tend to have a higher risk of early cancer recurrence and shorter overall survival, while those with more subcutaneous fat (fat under the skin) and greater muscle density generally have better survival outcomes.

## 1. Introduction

Pancreatic cancer (PC) is one of the most lethal malignancies worldwide, characterized by poor prognosis and a high mortality rate, with the 5-year survival rate remaining low at around 5% [1]. The late-stage diagnosis, coupled with the tumour’s resistance to treatment, contributes significantly to the poor survival outcomes observed in PC patients. The most common subtype is pancreatic ductal adenocarcinoma (PDAC), which arises from the ductal cells lining the pancreatic ducts. PDAC is known for its aggressive nature and poor prognosis, often presenting at an advanced stage due to subtle early symptoms. Currently, 30–35% of patients present with locally advanced disease and 50–55% with metastatic disease at diagnosis [2]. Between 1990 and 2021, the global number of incident cases increased by 168.7%, and deaths increased by 168.2%, with disability-adjusted life years increasing by 148.5% during this same period [3]. This alarming trend is driven by population aging, increasing prevalence of modifiable risk factors such as obesity, diabetes, and smoking, and early-onset pancreatic cancer becoming more prevalent, particularly among younger women [4].

Cancer patients undergo many imaging examinations during their journey to fight cancer, including most frequently computed tomography (CT) and magnetic resonance imaging (MRI) [5,6,7,8,9]. Computed tomography (CT) is the most effective and widely used imaging modality for assessing body composition in patients with pancreatic cancer, primarily because it is routinely performed for staging and follow-up, and allows for precise quantification of skeletal muscle and adipose tissue compartments, including muscle quality (e.g., myosteatosis) and visceral adiposity [10]. In fact, from these imaging exams, it has been demonstrated that an opportunistic evaluation of many metrics can be performed, to add valuable information on the global status of the patient, including a comprehensive body composition assessment [11,12]. This opportunistic assessment of routinely acquired imaging represents a cost-effective approach to extract prognostic information without requiring additional testing or radiation exposure. CT-based analysis is highly reproducible, can be automated with artificial intelligence, and provides detailed regional information relevant to cancer cachexia and prognosis.

Body composition (BC) refers to the proportion of the main tissue components of the body (fat, muscle and bone). The BC evaluation, based on imaging examinations such as CT, may include quantitative assessment of muscle mass by skeletal muscle area (SMA) and skeletal muscle index (SMI), as well as assessment of fat distribution as subcutaneous adipose tissue (SAT) and visceral adipose tissue (VAT) [13]. Additionally, muscle quality can be assessed through skeletal muscle radiodensity (SMD) or skeletal muscle attenuation (SMA), with lower values indicating myosteatosis (fat infiltration in muscle) [14]. There is a growing literature showing associations between body composition assessment and prognosis in many cancer subtypes, including ovarian [15], lung [16] and bladder [17] cancer. Unlike body weight or body mass index (BMI), that provide nonspecific measurements, imaging-based BC assessment offers a more detailed understanding of health by distinguishing between lean mass, including muscles and bones, and fat mass. This distinction is crucial, as it influences metabolic functions, physical performance, and overall health. Notably, studies have demonstrated that normal or even elevated BMI may mask significant muscle depletion, a phenomenon known as “hidden sarcopenia” or sarcopenic obesity, which affects prognosis independently of body weight [14].

Indeed, a reduction in lean body mass, also referred to as sarcopenia, is highly prevalent in pancreatic cancer patients, affecting approximately 30–65% of patients at diagnosis depending on the diagnostic criteria used. [18]. The pooled prevalence across studies is 45% when measured using skeletal muscle index, making pancreatic cancer the malignancy with one of the highest rates of sarcopenia [19]. This muscle depletion occurs due to the combined effects of systemic inflammation, malnutrition, and tumor-related metabolic disturbances [20]. Pancreatic cancer-associated cachexia, a multifactorial syndrome encompassing sarcopenia, weight loss, anorexia, and metabolic derangement, affects over 80% of pancreatic cancer patients, the highest rate among all malignancies, contributing to reduced quality of life, decreased tolerance to treatment, and significantly reduced survival. The relationship between changes in BC and survival in PC is therefore of clinical interest, as it may help identify patients at higher risk of poor outcomes and might guide personalized interventions [21]. Recent evidence demonstrates that sarcopenia at diagnosis is an independent prognostic factor, with meta-analyses showing hazard ratios of 1.42 for overall survival and 1.41 for progression-free survival [22]. Furthermore, longitudinal changes in muscle mass during treatment appear equally or more important than baseline measurements, with studies showing that patients experiencing greater muscle loss during chemotherapy have significantly worse outcomes regardless of initial muscle status [23]. Beyond muscle mass, other body composition parameters including visceral adiposity (particularly visceral-to-subcutaneous fat ratio), myosteatosis, and their longitudinal changes have emerged as independent predictors of survival, suggesting that a comprehensive assessment of multiple body composition compartments may provide more nuanced prognostic information than any single parameter [24]. Understanding how BC affects the survival of PC patients could be crucial for improving prognostication, informing patients about treatment strategies, and enhancing their care. This systematic review aims to critically analysing and synthesizing the existing literature, in order to provide a clearer understanding of whether BC measurements extracted from CT scans, can serve as reliable prognostic indicators for PC outcomes. Given the high prevalence of body composition abnormalities in pancreatic cancer, the availability of routine imaging for opportunistic assessment, and the growing evidence for their prognostic significance, integrating body composition analysis into clinical practice may facilitate earlier nutritional interventions, better risk stratification, and personalized treatment planning to ultimately improve patient outcomes.

## 2. Materials and Methods

### 2.1. Search Strategy

The research was done in compliance with the PRISMA guidelines (see Supplementary Table S1). The review was registered with the INPLASY with the registration code INPLASY202610026. To ensure comprehensive coverage of relevant literature, the databases PubMed and SCOPUS were screened for studies published between 2014 and 2024, with search strings and terms tailored to each database. Details of the screening progress are available in the Appendix A.

### 2.2. Selection Criteria

The following inclusion criteria were applied: (1) publications written in English; (2) available full free text and/or open access; (3) adults only. Exclusion criteria were as follows: (1) studies that did not present original data; (2) non-CT-based body composition assessment; (3) focus on other cancer types; (4) animal studies.

### 2.3. Data Extraction

For each eligible article, information was collected enabling comparisons based on factors such as basic study data, including authors, country of origin, year of publication, and the study design being prospective or retrospective; population characteristics, such as number of patients, sex, age and sarcopenic status; body composition features evaluated were extracted and included: skeletal muscle (SKM), VAT, SAT, muscle mass (MM), SMI, mean muscle attenuation (MMA), total adipose tissue (TAT), intramuscular adipose tissue (IMAT), visceral to muscle ratio (VMR), visceral to subcutaneous adipose tissue area (VSR), subcutaneous fat area (SFA), visceral fat area (VFA), subcutaneous fat area (SFA), total fat mass (TFM), total fat area (TFA), skeletal muscle density (SMD), skeletal muscle radiodensity (SMRD), fatty muscle fraction (FMF), intramuscular fat fraction (IMFF), visceral adipose tissue index (VATI), and subcutaneous adipose tissue index (SATI); association of body composition features and survival. After extraction, we realized that it was difficult to compare features that were named in different ways but indicated the same assessment. For this reason, we secondarily grouped some features to ensure comparisons, as detailed in the results Section (see Results Section 3.3).

### 2.4. Quality Assessment

The overall quality of the included studies was critically evaluated based on the revised “Quality Assessment of Diagnostic Accuracy Studies” tool (QUADAS-2) [25]. This tool comprises four domains (patient selection, index test, reference standard, and flow and timing) and each domain was assessed in terms of risk of bias, and a graph was constructed appropriately. More in details, in the patient selection domain, it was examined how study participants were chosen, including inclusion/exclusion criteria, recruitment methods (consecutive, random, or convenience sampling), and whether inappropriate exclusions were made, with the aim of assessing whether the patient spectrum matched the intended clinical population and if selection methods could have introduced bias. In the index test domain, we evaluated the conduct and interpretation of the body composition parameters included in the papers, if the parameters were standardized, and whether the test threshold was pre-specified. In the reference standard domain, we evaluated the presence and appropriateness of a reference standard. In the flow and timing domain, we examined the sequence and timing of the index test and reference standard and whether all patients were included in the analysis.

## 3. Results

### 3.1. Study Selection Process

**Figure 1:**
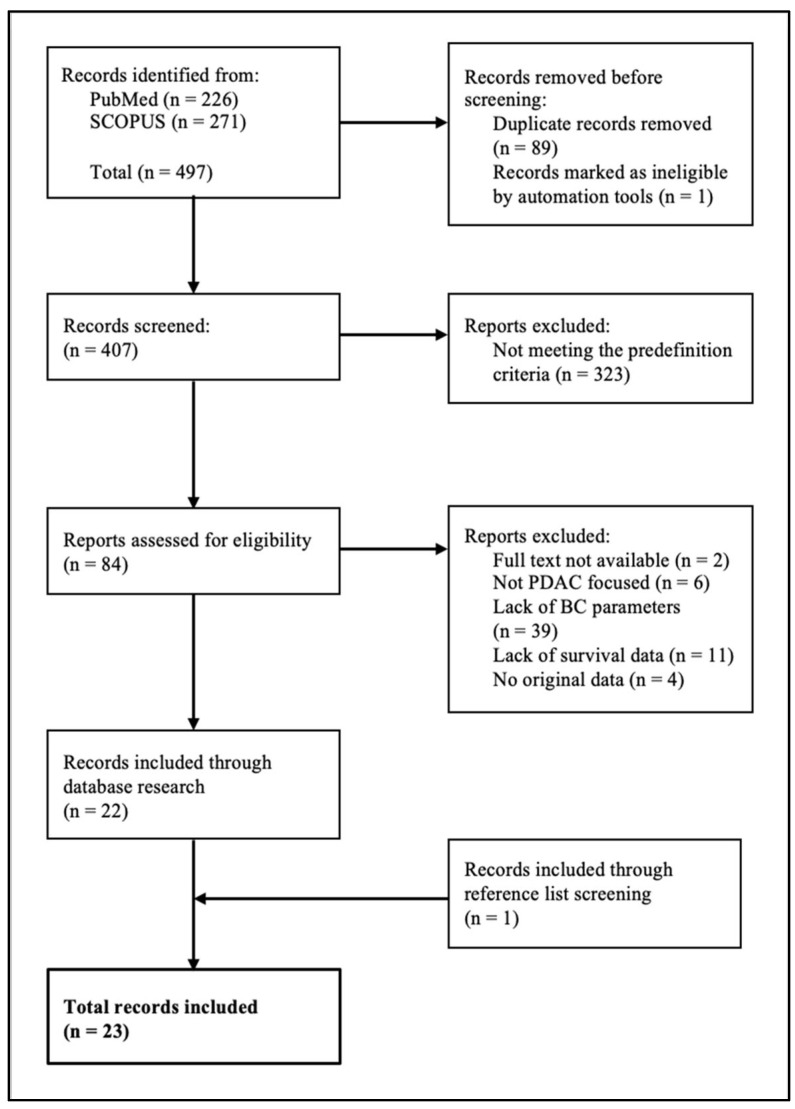
Flowchart for screening literature.

The database research resulted in 497 findings. According to the inclusion and exclusion criteria, 23 studies were included in this review (Figure 1).

### 3.2. Characteristics of Included Studies

All studies [21,26,27,28,29,30,31,32,33,34,35,36,37,38,39,40,41,42,43,44,45,46,47] were conducted in high- and middle-income countries with the majority being retrospective in design (3/23; 13%). Sample size ranged from 53 [34] to 476 patients [39], with only one study including more women than men.

The reported mean age of participants ranged from 59.5 to 74.6 years across the included studies. All studies reported the mean or median BMI of participants, which ranged from 21.0 to 28.7 kg/m^2^. The percentages of sarcopenic patients were not available in all studies. In those available they ranged from 32.2 [27] to 65.1% [47].

### 3.3. Technical Aspects and Features Evaluated

All 23 studies used diagnostic CT scans at the L3 vertebral level for body composition analysis. Since the included studies used different definitions and cut off values for their measurements and calculations, as well as for definition of sarcopenia, we grouped some of them to facilitate the comparison among the different body components. More in details: fatty muscle infiltration (FMF) included FMF, IMAC, IMAT, IMATI, IMFA, IMFF; muscle attenuation (MA) included MA, MD, MMA, PMD, RA, SMD, SMRD; muscle mass (MM) included PMA, SKM, SMA; skeletal muscle indices (SMI), usually indicating the skeletal muscle area divided by the square height, included SMI, ASMAH, PMAH, PMTH, TPI; total adiposity (TA) included TA, TAT, TFA, TFM; subcutaneous adipose tissue (SAT) included SAT and SFA; subcutaneous adipose tissue index (SATI), usually indicating the subcutaneous adipose tissue divided by the square height, included SATI and SFI; visceral adipose tissue (VAT) included VAT and VFA; and visceral adipose tissue index (VATI), usually indicating the visceral adipose tissue divided by the square height, included VATI and VFI.

Twenty-one studies reported an association of features accounting for skeletal muscle and adipose tissue with survival (Table 1). 21/23 studies found a significant association between at least one BC parameter and OS. Two studies did not find a significant association between standard BC metrics and survival [27,42], but Damm et al. noted myosteatosis as a significant prognostic factor [27], and Bareiss et al. highlighted the nutritional risk score (NRS) as a predictor of survival [42], thus confirming that the body composition, although evaluated by different metrics, was still important for survival. Eight studies [29,30,31,32,33,41,42,43,44] mentioned a correlation of low baseline SMI (referring to it as sarcopenia) and shorter OS, although some of these findings were limited to specific subgroups based on sex or BMI thresholds. Two studies [36,44] further noticed that the correlation became even stronger if a high VAT was present. Four studies [35,36,46,47] found a correlation between low SMI and shorter overall survival only in the presence of high adipose tissue, indicating that not sarcopenia alone, but sarcopenic obesity, was associated with reduced OS. Visceral adiposity showed mixed effects across studies: while four studies [34,36,39,44] reported shorter OS in patients with high baseline VAT, one study [21] found greater OS associated with high VAT. Subcutaneous adiposity was mentioned by two studies [21,39], both reporting a higher SAT/SATI correlating with greater OS. Eleven studies [21,26,28,33,34,37,40,41,43,45,46] reported that changes in one or more of the body composition parameters during treatment were predictive of OS, with some emphasizing that these dynamic changes were more informative than baseline values alone. These results underline the growing consensus that body composition is a potential meaningful predictor of survival in pancreatic cancer patients. However, it also shows variability in methodology and findings, particularly in the relative importance of each parameter, as well as the timing (and naming) of their measurement, and their monitoring.

### 3.4. Quality Assessment Results

**Figure 2:**
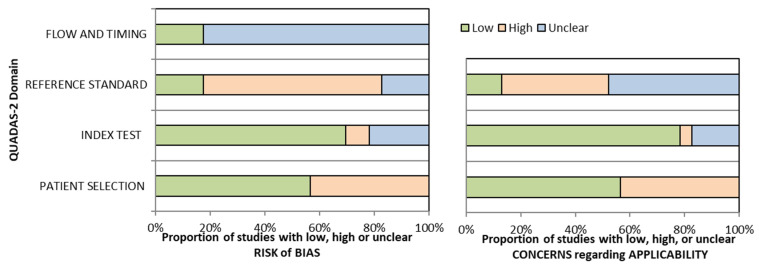
Overall Quality Assessment of the Studies Included, according to the QUADAS-2 Tool.

The overall quality assessment of the studies is reported in Figure 2.

## 4. Discussion

This systematic review included 23 studies that investigated the association between CT-based body composition and overall survival in patients with pancreatic cancer. In this systematic review, we included only CT as imaging method to assess body composition because CT remains the gold standard for body composition analysis in pancreatic cancer due to its precision, accessibility, and ability to assess both quantity and quality of muscle and adipose tissue. Dual-energy X-ray absorptiometry (DXA) is a validated alternative for whole-body composition assessment, offering accurate measurements of fat mass and lean mass in the general population and in cancer patients. However, DXA is less effective than CT for quantifying regional muscle and visceral adipose tissue, and is not interchangeable with CT in cancer populations, including pancreatic cancer survivors, due to systematic biases and limited ability to distinguish tissue compartments affected by cancer or treatment [48]. DXA estimates of visceral adipose tissue show high correlation with CT, but substantial bias exists, especially in patients with prior abdominal surgery or ascites, limiting its clinical utility for this population. Bioelectrical impedance analysis (BIA) is less accurate than both CT and DXA, with significant under- or overestimation of fat-free mass and poor agreement in cancer patients [48]. Magnetic resonance imaging (MRI) offers similar accuracy to CT but is less accessible, not routinely used for body composition in pancreatic cancer patients and more prone to technical issues for comparisons.

As technical aspect of body composition evaluation on CT scanners, the third lumbar vertebra (L3) is the standard anatomical landmark used in the vast majority of studies for body composition analysis and image protocols. This level is chosen because single-slice measurements at L3 correlate strongly with whole-body muscle and adipose tissue volumes [49]. Although images without or with contrast medium may be used for body composition assessment from CT scans, in the specific setting of pancreatic cancer, portal venous phase images at the L3 level are the most commonly analyzed.

Eight studies [29,30,31,32,33,41,42,43,44] mentioned a correlation of sarcopenia and shorter OS, although some of these findings were limited to specific sub-groups based on sex or BMI thresholds. Four studies [35,36,46,47] found a correlation between low SMI and shorter OS only in the presence of high adipose tissue, indicating that more than sarcopenia alone, sarcopenic obesity, was associated with reduced OS. Similar results were demonstrated in patients candidate to surgery for PC [50]. Indeed, some studies found a correlation between sarcopenia and survival, others did not report a correlation, thus resulting in unequivocal conclusions. These conflicting results may be partially explained by differences in treatment intention (curative versus palliative) and disease stage, as demonstrated by recent large-scale meta-analyses showing stronger associations between sarcopenia and survival in patients undergoing curative treatments (hazard ratio [HR] 1.53–1.62) compared to palliative settings [51]. The corresponding meta-analysis confirmed a reduced survival in patients with sarcopenia, whereas a clear correlation with short-term postoperative outcomes was not evident [50]. Accordingly, Liu et al. demonstrated that preoperative sarcopenia was significantly associated with poor prognosis in pancreatic cancer patients following radical surgery, but they underlined that this association would require further validation through prospective studies [52].

Large-scale cohort studies and prospective clinical trials have provided more definitive evidence. A recent meta-analysis of 48 studies involving 9063 patients demonstrated a pooled sarcopenia prevalence of 45% in pancreatic cancer, with significant prognostic impact on both overall survival (pooled adjusted HR 1.39, 95% CI 1.16–1.66) and progression-free survival (pooled adjusted HR 1.31, 95% CI 1.11–1.55) [53].

Importantly, one of the first prospective multicenter randomized trials examining body composition in 90 patients with resectable pancreatic adenocarcinoma receiving neoadjuvant chemotherapy found that visceral adipose tissue area (HR 1.58, 95% CI 1.00–2.51, *p* = 0.05) was associated with survival, but surprisingly found no significant difference in median overall survival between patients with versus without sarcopenia (23.6 versus 27.9 months, *p* > 0.05) [54]. This suggests that the prognostic impact of body composition parameters may differ significantly between early-stage resectable disease and advanced disease, highlighting the need for stage-specific and treatment-specific validation of body composition markers.

Visceral adiposity showed mixed effects across studies, with some studies reporting shorter OS in patients with high baseline VAT [34,36,39,44], and one study [21] showing longer OS associated with high VAT. On the other hand, SAT and SATI correlated with longer OS [24,39].

Visceral fat is more metabolically active, secreting higher levels of pro-inflammatory cytokines and adipokines, and is closely associated with systemic inflammation, insulin resistance, and a tumor-promoting microenvironment. Its anatomical proximity to the pancreas facilitates direct crosstalk between adipocytes and cancer cells, promoting invasion, metastasis, and aggressive tumor behavior. Increased visceral fat density, which may reflect fibrosis, inflammation, or cachexia-related adipocyte shrinkage, is associated with shorter survival and higher risk of disease progression in pancreatic cancer. On the other hand, subcutaneous fat is less metabolically active and less involved in inflammatory signaling. Higher subcutaneous fat area is associated with longer survival in PC patients, potentially because it serves as an energy reservoir and is less susceptible to cancer-driven catabolism and cachexia. Patients with greater subcutaneous fat are less likely to experience rapid nutritional decline and may have better tolerance to cancer therapies [39].

Recent large multicenter studies have clarified these contradictory findings by demonstrating that the visceral-to-subcutaneous fat ratio (VSR), rather than absolute visceral fat quantity alone, is a more robust predictor, with high VSR independently predicting both early recurrence (OR 2.30, *p* = 0.001) and worse overall survival (HR 1.46, *p* = 0.007) in resectable pancreatic cancer [24]. Additionally, visceral adipose tissue density, rather than volume alone, has emerged as a novel predictive biomarker, with increased VAT density showing strong association with overall survival (*p* = 0.0019) across all pancreatic cancer subgroups (resectable, unresectable, and metastatic) [55].

The impact of adiposity, including subcutaneous fat and visceral fat, on survival in PC patients appears complex and context-dependent, with mixed findings across studies. Most importantly, many studies emphasized that not only baseline measurements but rather their development after diagnosis and during treatment hold prognostic value.

Large prospective cohort studies have confirmed that longitudinal changes in body composition may be equally or more important than baseline values. In a study of 456 patients with metastatic pancreatic cancer, body composition changes occurred predominantly during the first 2 months after starting chemotherapy, with change in subcutaneous adipose tissue index being significantly associated with overall survival in male patients (HR 0.51, 95% CI 0.35–0.75, *p* < 0.001), independent of baseline sarcopenia status [43].

Similarly, a study of 105 patients with unresectable pancreatic cancer demonstrated that early changes in skeletal muscle index (ΔSMI) at first follow-up were prognostic for overall survival (HR 1.2, 95% CI 1.08–1.33, *p* = 0.001) even after adjusting for RECIST response assessment, with patients experiencing lower rates of muscle loss showing median survival of 233 versus 143 days. Therefore, the relationship between baseline and overtime measurements requires more research, possibly in prospective randomized trials [23].

A deeper insight the top quality studies reporting data on correlations between adiposity and survival, shows that Damm et al. [27], including 354 patients and used standardized, sex-adjusted regression formulas derived from a healthy population, demonstrated that myosteatosis was the only independent prognostic factor among body composition parameters. Pecchi et al. [21] analysed both baseline and longitudinal changes in adiposity parameters showing that higher visceral fat index was associated with greater survival rates in Cox regression models.

Bian et al. [44] included 203 patients with advanced pancreatic cancer and used multivariable analyses controlling for performance status and tumor stage. Visceral-to-subcutaneous adipose tissue ratio was identified as an independent risk factor (HR 1.38, *p* = 0.005), demonstrating the importance of fat distribution over total adiposity.

Therefore, the relationship between baseline and overtime measurements requires more research, possibly in prospective randomized trials.

Furthermore, several studies in this review suggest that a combined effect of sarcopenia and adiposity, commonly referred to as sarcopenic obesity, does exist. Three studies specifically found that sarcopenic obesity was significantly associated with poorer OS compared to either condition alone [35,46,47]. Additionally, two other studies reported that the negative impact of low muscle mass on survival became more pronounced in the presence of high visceral fat, suggesting a synergistic adverse effect [36,44]. Gruber et al. demonstrated in 133 patients with resectable PC that sarcopenic obese patients had significantly poorer overall survival compared to non-sarcopenic obese patients (14 versus 23 months, *p* = 0.007) and showed higher incidence of major postoperative complications (*p* < 0.001) [56]. These findings support the idea that evaluating muscle and fat compartments together provides a more accurate prognostic picture than assessing either in isolation. However, the definitions and thresholds for sarcopenic obesity varied among studies, limiting direct comparisons. This underscores the need for standardized criteria in assessing and reporting sarcopenic obesity in PC populations. Furthermore, sarcopenic obesity can be defined itself according to different definitions [57,58,59], but only two [35,38] of the included articles specified the definition of sarcopenic obesity they referred to [57,58].

Eleven studies [21,26,28,33,34,37,40,41,43,45,46] reported that changes in one or more of the BC parameters during treatment were predictive of OS, with some emphasising that these dynamic changes were more informative than baseline values alone. These results emphasize that BC measurements are a potential meaningful predictor of survival in cancer patients. However, it also reveals some variability in methodology and findings, particularly in the relative importance of each parameter, as well as the timing of their measurement and monitoring.

It should also be noted that more than half of the included studies showed high risk of bias or uncertain risk of bias, and several studies also raised concerns regarding applicability, therefore the degree of evidence of the results is downgraded, which means that the true effect of the body composition component may be substantially different from the review’s findings. In practice, this systematic review, with a predominance of high-risk studies may only be able to suggest associations rather than establish causality.

If a significant correlation between BC and survival is unanimously confirmed, BC assessments could be incorporated into routine clinical practice as a prognostic tool. This would allow healthcare providers to identify patients at higher risk of poor outcomes early, leading to more personalised handling. However, this review highlights several critical limitations in current research worldwide. The most significant limitation is the lack of standardized definitions for sarcopenia and adiposity across different populations. A recent comprehensive analysis applying 14 different published sarcopenia cutoff definitions to the same cohort of 179 patients found that prevalence ranged from 8.9% to 69.8%, with only 3 of 14 definitions showing significant association with overall survival [60]. Similarly, a systematic review and meta-analysis demonstrated that sarcopenia prevalence in pancreatic cancer varied from 19% to 57% depending on whether cutoff values were <40 cm^2^/m^2^, 40–50 cm^2^/m^2^, or >50 cm^2^/m^2^ [53]. This substantial variability undermines the comparability of studies and clinical applicability of findings. Furthermore, skeletal muscle mass and strength are significantly influenced by factors such as gender, ethnicity, body size, age, lifestyle, and cultural background, yet most studies have been conducted in high- and middle-income countries with predominantly White populations, restricting the generalizability of findings [61].

An additional major limitation is the predominance of retrospective study designs. While meta-analyses have synthesized data from thousands of patients, the vast majority of included studies are retrospective and observational. Of the 23 studies included in this systematic review, involving 5888 patients, all were retrospective.

The limited number of prospective trials restricts our ability to establish causal relationships and optimal intervention strategies. Moreover, many studies focused on specific subgroups defined by disease stage or treatment type, which limits external validity and the ability to develop universal prognostic models applicable across the full spectrum of pancreatic cancer presentations.

Future research should aim to establish consensus criteria and standardised methods for assessing BC in PC patients, and more in general in cancer patients.

Recent efforts to apply artificial intelligence (AI) and deep learning to body composition analysis offer promising solutions to standardization challenges. Several studies have demonstrated that fully automated AI-based body composition analysis from routine staging CT scans can provide consistent, reproducible measurements with high accuracy (Dice coefficients ≥ 0.92–0.94), eliminating inter-observer variability inherent in manual or semi-automated methods [55].

A multicenter study of 601 pancreatic cancer patients demonstrated that deep learning-derived muscle-to-bone ratio (MBR) and myosteatosis were significantly associated with overall survival (MBR: HR 0.60, *p* < 0.005; myosteatosis: HR 3.73, *p* < 0.005) independent of age, sex, and AJCC stage, with the AI analysis automatically processing routine staging CT scans across different institutions [61]. This automated approach enables seamless integration into standard oncologic workflows without additional procedural burden or radiation exposure, transforming opportunistic imaging into actionable clinical data.

Future research directions should focus on several key areas. First, prospective randomized controlled trials are needed to validate body composition cutoffs and evaluate whether interventions targeting sarcopenia and cachexia can improve outcomes. Second, longitudinal studies examining temporal changes in body composition throughout the cancer care continuum—from diagnosis through treatment and survivorship—are essential to understand the dynamic nature of body composition changes and identify critical time windows for intervention. Third, investigation into the biological mechanisms linking body composition alterations to pancreatic cancer outcomes is crucial. Preliminary studies suggest that sarcopenia may worsen prognosis by suppressing local.

This review has some limitations. One is the limited external validity, as some studies focused on subgroups defined by disease stages or specific treatment. In addition, all studies were conducted in high- and middle-income countries, restricting the applicability of findings to populations with similar characteristics and lifestyles. However, the body composition, as well as the nutritional intake in these populations would be anyway not comparable with that of low-income countries. A second limitation is that the studies included in this review were of varied design, including observational studies, cohort studies and others. This diversity in design can lead to heterogeneity in findings, as different methodologies may introduce varying degrees of bias and confounding factors. An additional limitation is that although all studies selected used CT-based body composition assessment, the accuracy of these measurements may vary according to the CT equipment and protocols, as well as to the specific software used for the extraction. Furthermore, along with varying levels of precision, different tools can name the same measure differently, and some studies may emphasize one measure, without a comprehensive assessment of all the measurements available. In order to partially overcome these limitations, we analysed the measurements included in different papers and grouped under the same name those that measured the same (or almost the same) body compartment. This helped us to make consistent comparisons across studies. Another limitation relies on the lack of standardization in definition of sarcopenia and adiposity. Indeed, there is no universal threshold for defining these entities based on imaging, as skeletal muscle mass and strength are significantly influenced by factors such as gender, ethnicity, body size, age, lifestyle, and cultural background leading to inconsistencies in how BC was categorized across studies, thus leading to a limited generalizability to specific populations. To overcome this limitation, in order to compare results of different papers, we grouped the BC definitions in wider categories.

## 5. Conclusions

In conclusion, body composition parameters show promise as prognostic markers, with longitudinal changes appearing more predictive than single-point measurements. However, future research should prioritize prospective studies employing standardized definitions and validated thresholds in order to help integrate BC assessment into routine practice in pancreatic cancer care.

## Figures and Tables

**Table 1:** Key findings of included studies.

Authors	Sarcopenia in %	Body Composition Features Evaluated	Correlation Found	Main Association(s) of CT-Based Body Composition and Survival
[26]	63%	MM, VAT, SAT	Yes	Increase in SKM during postoperative period was associated with higher OS.
[27]	32.2%	MM, SMI, MA TA, SAT, VAT, FMF, VMR, VSR	No	Myosteatosis as an independent prognostic factor
[28]	63.1%	SAT, VAT, SMI	Yes	Muscle loss during treatment was a strong independent predictor of poor survival.
[29]	64.2%	SMI, SAT, VAT, TA	Yes	Sarcopenia was an independent prognostic factor for OS with a BMI > 22
[30]	41%	TA, SMA, SMI, MA	Yes	Low SMI and low MA were poor prognostic factors for OS
[31]	62%	SAT, VAT, SMI, MM	Yes	A sex-standardised SMI was a predictive factor for shorter OS and RFS.
[32]	NA	SMI, MA, FMF	Yes	Sarcopenia and myosteatosis showed a prognostic value for OS.
[33]	44.2%	SMI, VATI, SATI, VSR	Yes	SMI change rates presented a trend to poor prognosis
[34]	NA	TA, SAT, VAT, VATI, MA, SMI, FMF	Yes	High VATI at baseline and loss of SMI during treatment were predictors of poor OS.
[35]	49%	SMI, SAT, VAT	Yes	Sarcopenic obesity associated with poor prognosis.
[36]	63%	SMI, SAT, VAT	Yes	Sarcopenia and high VAT were associated with poor survival,
[37]	NA	SMI, SAT, VAT	Yes	Loss of SAT, VAT and SMI during therapy indicated poor OS.
[38]	NA	SMI, SAT, VAT, FMF	Yes	Low FMF was associated with reduced survival.
[39]	NA	SKM, SAT, VAT, SMI, SATI, VATI	Yes	High SAT was associated with longer OS; high VAT was associated with shorter PFS.
[40]	52%	SMI, SMA, SAT, VAT	Yes	Muscle loss after diagnosis was associated with low OS
[41]	33.3%	SMI, MA, SAT, VAT	Yes	Preoperative sarcopenia and accelerated muscle loss after surgery negatively impacted OS.
[42]	28%	MA, SMI, SAT, VAT, MM, VSR, VMR	No	The nutritional risk score was associated with OS.
[43]	33.8%	MA, SMI, VATI, SATI	Yes	Initial MA was a significant prognostic factor for both sexes. Initial SMI and changes in SATI were associated with OS in males; in females initial SATI was significant.
[44]	NA	SMI, VSR, SAT, VAT, MA	Yes	Low SMI and high VAT were associated with poor OS.
[45]	49.1%	SMI, SATI, VATI, VSR	Yes	High loss in VATI was an independent risk factor for mortality.
[21]	56.8%	TAMA, SMI, FMF, SATI, VATI	Yes	High VATI and SATI correlated with greater OS. High VATI loss correlated with worse OS.
[46]	49.1%	SMI, FMF, VAT, VATI, SAT, SATI, TA, TAI	Yes	Sarcopenic obesity at diagnosis was associated with decreased OS. Adipose tissue and muscle loss during treatment was associated with decreased OS.
[47]	65.1%	TAMA, SAT, VAT	Yes	Sarcopenic obesity was associated with decreased OS.

## Data Availability

No new data were created or analyzed in this study.

## Supplementary Materials

The following supporting information can be downloaded at: https://www.mdpi.com/article/10.3390/tomography12010008/s1, Table S1: the PRISMA guidelines for abstract checklist. Ref. [62] is cited in Supplementary Materials.

## Abbreviations

The following abbreviations are used in this manuscript:

BC	Body Composition
BIA	Bioelectrical impedance analysis
BMI	Body Mass Index
CT	Computed Tomography
DXA	Dual-energy X-Ray Absorbiometry
FMF	Fatty Muscle Fraction
IMAT	Intramuscular Adipose Tissue
IMFF	Intramuscular Fat Fraction
L3	Third lumbar vertebra
MA	Muscle Attenuation
MM	Muscle Mass
MMA	Mean Muscle Attenuation
MRI	Magnetic Resonance Imaging
OS	Overall Survival
PC	Pancreatic Cancer
PDAC	Pancreatic Ductal Adenocarcinoma
PFS	Progression Free Survival
SAT	Subcutaneous Adipose Tissue
SATI	Subcutaneous Adipose Tissue Index
SKM	Skeletal Muscle
SMA	Skeletal Muscle Area
SMD	Skeletal Muscle Density
SMI	Skeletal Muscle Index
SMRD	Skeletal Muscle Radiodensity
TA	Total Adiposity
TAI	Total Adipose Index
TAMA	Total Abdominal Muscle Area
TAT	Total Adipose Tissue
TFA	Total Fat Area
TFM	Total Fat Mass
VAT	Visceral Adipose Tissue
VATI	Visceral Adipose Tissue Index
VMR	Visceral to Muscle Ratio
VSR	Visceral to Subcutaneous Adipose Tissue Area

## Appendix A

### Screening Details

The last screening for this systematic review was performed on the 17 January 2025 for PubMed and on the 20 January 2025 for Scopus. The used search strings were as follows: [“Pancreatic Neoplasms”[Mesh] OR “pancreatic cancer” OR “pancreatic tumour” OR “pancreatic carcinoma”] AND [[“Body Composition”[Mesh] OR “body composition” OR “muscle mass” OR “sarcopenia” OR “adiposity” OR “fat mass” OR “lean body mass” OR “BMI” OR “body mass index”] AND [“Survival Analysis”[Mesh] OR “survival” OR “prognosis” OR “survival rate” OR “mortality” OR “outcome”]] for PubMed and TITLE-ABS-KEY[“pancreatic cancer”] AND TITLE-ABS-KEY[“body composition” OR “sarcopenia” OR “muscle mass” OR “fat mass” OR “adiposity” OR “BMI”] AND TITLE-ABS-KEY[“outcomes” OR “survival” OR “prognosis” OR “treatment response”].
